# A Surgical Handover System for Patient Physiology and Safety

**DOI:** 10.1001/jamanetworkopen.2025.38896

**Published:** 2025-10-06

**Authors:** Jessica M. Ryan, Therese M. Lynn, Dara O. Kavanagh, Jan Sorensen, Anastasija Simiceva, Walter Eppich, Barry O’Sullivan, Alexandra Zaborowski, Tom V. McIntyre, Gerard F. Curley, Bridget Egan, Saoirse Morrin, XinYi Low, Joel Rajesh, Frank Crossen, David Hearne, Alexandra Troy, Sri Qistina Emily Mohammad Feisal, Caelan Mulligan, Laura Labbe, Angelyn Chow Pui Shan, Ian S. Reynolds, Helen Earley, Deborah A. McNamara

**Affiliations:** 1 Royal College of Surgeons in Ireland School of Postgraduate Studies, Dublin, Ireland; 2 Department of Surgical Affairs, Royal College of Surgeons in Ireland, Dublin, Ireland; 3 The Bon Secours Hospital, Dublin, Ireland; 4 Tallaght University Hospital, Dublin, Ireland; 5 Department of Surgery, Tallaght University Hospital, Dublin, Ireland; 6 Royal College of Surgeons in Ireland Healthcare Outcomes Research Centre, Dublin, Ireland; 7 Faculty of Medicine, Dentistry and Health Sciences, University of Melbourne, Melbourne, Australia; 8 Department of Surgery, Beaumont Hospital, Dublin, Ireland; 9 Royal College of Surgeons in Ireland Education & Research Centre, Beaumont Hospital, Dublin, Ireland; 10 School of Medicine, Trinity College Dublin, Dublin, Ireland; 11 School of Medicine, Royal College of Surgeons in Ireland University of Medicine and Health Sciences, Dublin, Ireland; 12 Office of the President, Royal College of Surgeons in Ireland, Dublin, Ireland

## Abstract

**Importance:**

Ineffective patient handover leads to patient harm, yet no criterion standard exists for safe and effective practice in surgery.

**Objective:**

To determine whether the SIPS (sickest patients first; introduction, situation, background, assessment, recommendation; prioritize; summarize) surgical handover system is associated with improved patient physiology and safety.

**Design, Setting, and Participants:**

This prospective interventional cohort study included an effectiveness-implementation hybrid design and was carried out between January 2023 and June 2024 at the general surgery departments of 2 tertiary academic hospitals. Physicians participating in postcall (emergency) general surgery handover meetings were included. Data were collected for consecutive patients admitted for emergency general surgery before and after implementation of the intervention, providing they had a minimum of 6 hours of Early Warning Score data available following the time of the handover meeting. Data were analyzed from November 27, 2023, to May, 8, 2025.

**Exposure:**

Staff were trained in the use of a 4-step approach to handover meetings, SIPS, which defines the minimum steps required for safe surgical handover.

**Main Outcomes and Measures:**

Handover quality, changes in vital signs, length of stay, mortality, escalations in care, staff perceptions of safety, and implementation success were evaluated through handover observations, a retrospective review of patient records, and staff surveys.

**Results:**

Data were collected for 2261 patients, including 1469 patients before the intervention (708 [48.2%] female; mean [SD] age 54.6 [20.3] years) and 792 patients after the intervention (411 [51.9%] female; mean [SD] age 52.8 [20.6] years). A total of 182 residents took part in handovers during the study period, during which time 126 handover meetings were observed. After the intervention, handover quality improved across multiple domains without prolonging meeting duration and was associated with significant improvements in patient vital signs at 12 hours (170 patients [21.5%] vs 247 patients [16.8%]; difference, 4.6 [95% CI, 1.2 to 8.1] percentage points; *P* = .007) and 24 hours (212 patients [26.8%] vs 294 patients [20.0%]; difference, 6.7 [95% CI, 3.0 to 10.4] percentage points; *P* < .001). Staff-reported handover-related patient safety events also decreased after the intervention (13 days with events [19.7%] vs 4 days with events [4.6%]; difference, −15.1 [95% CI −4.5 to −25.6] percentage points; *P* = .004), with improvements in staff-perceived handover safety and quality. Successful implementation was confirmed by high rates of adoption, fidelity, and sustainability.

**Conclusions and Relevance:**

In this cohort study, implementation of the SIPS surgical handover system was associated with improvements in handover quality, patient physiology, and staff perceptions of safety without prolonging handover meetings.

## Key Points

**Question:**

Is the use of the SIPS (sickest patients first; introduction, situation, background, assessment, recommendation; prioritize; summarize) surgical handover system associated with improvements in patient physiology and safety?

**Findings:**

In this cohort study of 2261 patients, 126 handover meetings, and 182 staff members, implementation of the SIPS surgical handover system was associated with improved handover quality, patient vital signs at 12 and 24 hours, and staff-reported patient safety events.

**Meaning:**

This study found that the use of the SIPS surgical handover system was associated with improved early patient physiology and reduced handover-related patient safety events.

## Teaser

This cohort study describes patient and staff-reported outcomes before and after implementation of the SIPS surgical handover system (sickest patients first; introduction, situation, background, assessment, recommendation [ISBAR]; prioritize; summarize) in 2 tertiary academic hospitals.

## Introduction

Communication failures in health care remain a leading cause of patient harm and death,^1^ despite being largely preventable.^2^ Clinical handover involves transferring patient information and responsibility among clinicians,^3^ and is one of the most common communication events in health care.^4^ When carried out ineffectively, omissions of critical information and other errors can lead to serious patient harm.^5,6,7^

Efforts to improve shift-to-shift surgical handover date back to at least 2005,^8^ but despite available guidance, no widely accepted protocol exists.^8,9^ Standardization of handover processes has proven effective in other settings, resulting in fewer omissions of information in operating room (OR) to intensive care unit (ICU) transitions^10^ and fewer medical errors and preventable adverse events in pediatric handovers.^11^ However, transfer of these approaches to surgical specialties remains challenging. Acute surgical services manage patients with highly variable conditions and care pathways, many of whom require urgent, high-risk procedures. Handover periods often coincide with competing clinical demands, such as emergency procedures and elective operating lists, creating intense time pressure.^12^ These features distinguish surgical handovers from those in many medical specialties and can limit the applicability of protocols developed for other settings.

Clinicians typically overestimate their handover skills,^13^ an egocentric bias leading to the erroneous belief that information has been received and understood as intended.^14^ While innovations in surgical handover have focused largely on electronic tools and documents,^8^ handover is fundamentally a conversation^15,16,17,18^ that should be supported, but not replaced, by these resources. Successful interventions in other settings systematically promote 2-way communication and explicit confirmation of shared understanding.^11,19^ Another method of promoting a shared mental model^20^ and situational awareness includes highlighting priority patients and tasks for the coming shift.^16,17,18,21,22^ Although emphasizing the surgical patients with the most severe illness during handover shows promise, this approach has yet to be rigorously evaluated.^8^

Small-scale projects to improve components of surgical handover are frequently reported, variably implemented, and rarely sustained,^8^ leading to change fatigue and little evidence of impact.^23,24^ Our previous work demonstrates a compelling need for a standardized, whole-of-handover approach, one that addresses the entire handover meeting from start to finish and calls for rigorous assessment of its clinical effectiveness.^8,12,25^ In this study, we define the minimum steps required for safe surgical handover based on existing evidence, implement a comprehensive surgical handover system in 2 tertiary academic hospitals using a prospective interventional cohort design, and evaluate clinical effectiveness and implementation outcomes.

## Methods

This cohort study was reviewed and deemed exempt by the Tallaght University Hospital institutional review board; therefore, this study was registered with the quality and audit departments at both sites. All activities were carried out in accordance with institutional guidelines and maintaining strict data protection policies. In accordance with Irish and European law regarding the conduct of clinical audit, explicit consent was not required from patients.^26^ The Data Protection Officer in each site approved the process for collection of patient data. Identifiable data were pseudoanonymized and stored in a password-protected database. Use of the new handover process was mandated as part of an emergency surgical care improvement initiative based on previous institutional findings,^25^ with support from all relevant clinical leads. All staff surveys were anonymous, and survey completion was understood to indicate consent for participation. This study used a hybrid effectiveness-implementation design to evaluate the clinical effectiveness of a surgical handover system and its implementation.^27^ This study is reported following the Strengthening the Strengthening the Reporting of Observational Studies in Epidemiology (STROBE) reporting guideline for cohort studies.

### Design of the Intervention

**Figure 1.**
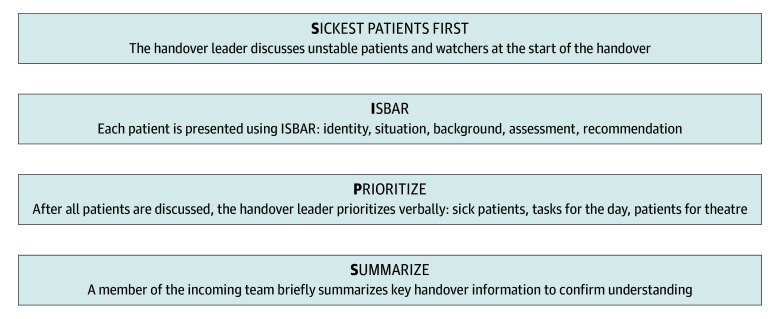
The SIPS Surgical Handover System

The SIPS surgical handover system (sickest patients first; introduction, situation, background, assessment, recommendation [ISBAR]; prioritize; summarize) was designed based on a comprehensive, mixed-methods evaluation of practice,^25^ a systematic review of previous interventional studies^8^ and a national survey of stakeholders.^12^ This novel, 4-step approach to surgical handover meetings (identified by the acronym SIPS) defines the minimum steps required for safe surgical handover (Figure 1). Step 1 is to identify the sickest patients at the outset. Step 2 uses a recognized framework, ISBAR, to present each patient.^28,29^ At step 3, participants identify high-priority patients and tasks for the upcoming shift. Step 4 concludes the process with a handover summary by the receiving team to confirm understanding.

The intervention was applied during structured handover meetings between outgoing and incoming surgical teams, during which the on-call patient list was reviewed, discussed, and responsibility for patient care was transferred. Each step of the intervention is assigned to a designated staff member to support implementation and facilitate coaching.

### Study Design

To test the effectiveness of this handover system, a prospective quasiexperimental interventional cohort study was conducted in 2 hospital sites using a before and after design. Staff employed in each hospital prior to the implementation of the SIPS surgical handover system served as the control group, followed by a separate cohort of staff trained in the new method who served as the intervention group. The SIPS surgical handover system was implemented in 4 stages guided by the Active Implementation Framework (eTable 1 in Supplement 1).^30^

### Setting and Staff Population

This study was carried out between January 9, 2023, and June 19, 2024, in the general surgical departments of 2 tertiary academic hospitals in Dublin, Ireland. Surgical team members who participated in the morning (postcall) emergency general surgery handover included interns, residents, and attendings. These grades are equivalent to interns, senior house officers, registrars, and consultants in the United Kingdom and Ireland. Prior to the intervention, neither site followed a standardized handover process; rather, the attending or senior resident on duty led handovers according to personal preference. Prior observational research at these sites revealed that overall adherence to best practice guidelines was poor.^25^

### Patient Population

Data were collected for all emergency general surgical patients admitted before and after implementation of the intervention. Patients were eligible for inclusion if they had a minimum of 6 hours of Early Warning Score (EWS)^31^ data available following the handover meeting. Patients admitted during the early implementation phase of the study were excluded from analysis as introduction of the intervention was still under way during this time (eTable 1 in Supplement 1).

### Outcome Assessment and Data Collection

Both clinical effectiveness and implementation outcomes were assessed (eTable 2 in Supplement 1). In brief, clinical effectiveness was evaluated through the assessment of handover quality, patient outcomes, and staff experience of handover, guided by prior work on handover outcome taxonomy.^8^ Implementation was assessed through the evaluation of adoption, fidelity, sustainability,^32^ and staff-reported acceptability, appropriateness, and feasibility^33^ of the intervention. A composite fidelity score was calculated based on adherence to core components of the intervention (range 0-10; higher score indicates better adherence) (eTable 3 in Supplement 1).

#### Handover Observations

Handover observations by research nurses, retrospective review of patient records, and staff surveys were used to assess clinical effectiveness and implementation outcomes. Trained research nurses carried out overt, random,^34^ nonparticipant observations of morning, weekday, handover meetings. Observers used a structured form (eAppendix 1 in Supplement 1) to assess fidelity and handover quality, uploading each observation to an online database, using previously described methods.^25^ Research nurses completed 4 hours of structured training based on best practices for rater preparation, including performance dimension training, frame-of-reference training, and behavioral observation training.^35,36,37,38^ A 1-on-1 online session was delivered by the study coordinator (J.M.R.), followed by at least 5 co-observed handover sessions, with in-person debriefing after each and additional training if required. Training effectiveness was confirmed by assessing interrater reliability between the study coordinator and research nurse prior to independent observations.^39^ Regular interim reviews were conducted with participating research nurses throughout the study to address any issues prior to the next scheduled observation.

#### Retrospective Review of Patient Records

Patient outcome data were obtained from hospital electronic patient records (for total length of stay, transfers to ICU, and mortality) and handwritten paper records (for EWS and vital sign data). Data regarding transfers to ICU and mortality were crosschecked against a prospectively maintained national database (the National Office of Clinical Audit–Intensive Care Unit database^40^). Individuals responsible for obtaining outcome data were not involved in the intervention.

#### Staff Surveys

A daily survey completed by interns and junior residents captured staff-reported handover-related patient safety events and any patients missed during the initial post-handover ward round. A handover-related patient safety event was defined as a patient safety issue which could have been prevented or ameliorated by the information shared during the morning handover. These events were categorized as near misses, negligible, minor, moderate, major, or extreme harm (eAppendix 2 in Supplement 1). When duplicate entries were submitted for the same day, those completed later or those without reported events (when another entry noted an event) were excluded from the analysis.

Because no standard instrument exists to evaluate staff experience of handover, a new survey was developed by reviewing previously published tools.^41,42,43,44,45,46,47,48,49^ A pilot version was administered to 1 representative of each junior staff grade and minor revisions were made based on their feedback. The final version of the survey was distributed to all junior staff (interns and residents) before and after implementation of the intervention to capture changes in perceptions of handover safety, efficiency, and quality (eAppendix 3 in Supplement 1). All items were rated on 5-point Likert scales.

### Statistical Analysis

Data were analyzed using Stata software version 18.5 (StataCorp) from November 27, 2023, to May, 8, 2025. Categorical data were presented as counts and percentages and continuous data as median (IQR) and mean (SD). Comparative analyses of quantitative data were performed using χ^2^ test for categorical data, *t* test, and Mann-Whitney *U* test for continuous data. Five-point Likert scales were coded numerically from 1 to 5, summarized using mean (SD), and analyzed using Welch 2-tailed *t* test for unequal variances.^50^ All tests of significance were 2-tailed, with *P* < .05 indicating statistical significance. The baseline EWS^31^ was defined as the most recent score documented before the handover meeting (designated as time 0). Subsequent scores at 6, 12, and 24 hours after handover were extracted for comparison.

## Results

Data were collected for 2261 patients, including 1469 patients before the intervention (708 [48.2%] female; mean [SD] age, 54.6 [20.3] years) and 792 patients after the intervention (411 [51.9%] female; mean [SD] age, 52.8 [20.6] years). During the postintervention period between November 2023 and April 2024, 98 handover meetings, encompassing 611 individual patient discussions, were observed, totaling 15.1 hours of direct observation across the 2 study sites. This was compared with baseline data collected between January and April 2023, which included 25 handover meetings and 211 patient discussions.^25^ A total of 110 junior physicians participated in handover during the postintervention period, compared with 72 physicians in the preintervention period. Almost all (99.1%) eligible staff completed training in use of the SIPS surgical handover system.

### Clinical Effectiveness Outcomes

#### Quality of Handover

Handover quality significantly improved postintervention, with an increase in attendees (median [IQR], 4 [3-4] attendees vs 5 [4-7] attendees; *P* < .001), sick patients highlighted at the outset (1 handover [4.0%] vs 73 handovers [98.6%]; difference, 94.6 [95% CI, 86.5 to 100] percentage points; *P* < .001), priority lists given (7 handovers [28%] vs 78 handovers [79.6%]; difference, 51.6 [95% CI, 32.2 to 71.0] percentage points; *P* < .001), and summaries provided (1 handover [4.0%] vs 85 handovers [86.7%]; difference, 82.7 [95% CI, 72.5 to 92.9] percentage points; *P* < .001). There was no change in the duration of handover meetings (median [IQR], 11 [7-14] minutes vs 8 [5-11] minutes; *P* = .09), and the number of patients discussed was also similar (median [IQR], 7 [5-11] patients vs 5 [4-8] patients; *P* = .08). Compared with before the intervention, junior staff were more likely to participate in morning handovers following the intervention (35 handovers [53.8%] vs 80 handovers [93.0%]; difference, 39.2 [95% CI, 25.9 to 52.5] percentage points; *P* < .001).

#### Patient Outcomes

Outcome data were available for 1477 patients in the preintervention period (May to November 2023) and 790 patients in the postintervention period (December 2023 to April 2024) (Table 1). A description of patients excluded from the analysis is available in Table 2. Mean length of hospital stay, the number of patients transferred to the ICU, and in-hospital mortality were similar before vs after the intervention.

**Table 1.** Demographics and Patient Outcome Data by Study Period

Characteristic	Patients, No. (%)		*P* value
	Preintervention	Postintervention	
Patients	1469 (65)	792 (35)	NA
Age, mean (SD), y	54.56 (20.28)	52.76 (20.60)	.046
Sex			
Female	708 (48.2)	411 (51.9)	.09
Male	761 (51.8)	381 (48.1)	
Length of hospital stay, mean (SD), d	7.65 (9.98)	7.69 (10.42)	.94
In-hospital mortality	12 (1)	6 (1)	.88
Transfers to ICU	41 (3)	20 (2)	.71
Baseline EWS			
Mean (SD)	0.78 (1.29)	0.83 (1.26)	.38
0-1	1211 (82)	644 (81)	.91
2-3	206 (14)	118 (15)	
4-6	42 (3)	25 (3)	
>6	10 (1)	5 (1)	
Improved EWS at 6 h	175 (11.9)	117 (14.7)	.05
Improved EWS at 12 h	247 (16.8)	170 (21.5)	.007
Improved EWS at 24 h	294 (20.0)	212 (26.8)	<.001

Abbreviations: EWS, Early Warning score^51^; ICU, intensive care unit; NA, not applicable.

**Table 2.** Summary of Patient Inclusion and Exclusion Criteria

Reason	Patients, No.		
	Preintervention	Postintervention	Total
Patients excluded from analysis			
Overall	430	321	751
Early Warning Score document not available	254	213	467
No baseline score at time of handover	2	2	4
<6 h of Data available after handover	174	106	280
Patients included in analysis	1469	792	2261
Total	1899	1113	3012

In contrast, the proportion of patients with improved vital signs, reflected by a reduction in EWS,^31^ was significantly higher after the intervention. At 12 hours after handover, an improvement in vital signs was observed in 170 postintervention patients (21.5%) compared with 247 preintervention patients (16.8%) (difference, 4.6 [95% CI, 1.2 to 8.1] percentage points; *P* = .007). This difference was more pronounced at 24 hours (212 patients [26.8%] vs 294 patients [20.0%]; difference, 6.7 [95% CI, 3.0 to 10.4] percentage points; *P* < .001).

Following the intervention, staff reported fewer handover-related patient safety issues. Daily staff surveys were returned on 66 eligible preintervention days (84.6%) and 86 eligible postintervention days (97.7%), with 25 duplicate entries excluded from the analysis. After the intervention, the proportion of days on which junior staff reported a patient safety event was significantly lower (13 days [19.7%] vs 4 days [4.6%]; difference, −15.1 [95% CI, −4.5 to −25.6] percentage points; *P* = .004).

#### Staff Experience of Handover

Staff experience of the handover process significantly improved after the intervention. A total of 40 survey responses (56.9%) were received before the intervention and 82 responses (85.8%) were received after the intervention, with comparable representation of hospital site and staff grade (eTable 4 in Supplement 1). Among junior staff, the handover was more frequently used to prioritize tasks for the upcoming shift after the intervention (Table 3). Staff also reported fewer instances of missing or incorrect information at handover, fewer patients who turned out to be sicker than anticipated, and fewer instances where patient care was negatively impacted due to handover. Staff also rated the postintervention handover process higher in efficiency, quality, and safety (Table 3). Questions 5 to 12 had Cronbach α = .87, indicating good internal consistency of this 8-item scale for measuring perceived handover quality and safety.^50^

**Table 3.** Staff Experience of the Handover Process

Question^a^	Preintervention (n = 40)		Postintervention (n = 82)		*P* value
	No.	Mean (SD)	No.	Mean (SD)	
How often did you attend the post-call morning handover on days that your team were post-take? (Junior staff only)	31	3.97 (1.45)	63	4.55 (1.07)	.05
How often was the post-call handover used to prioritise jobs to be completed that day? (Junior staff only)	31	3.87 (1.17)	63	4.60 (0.77)	.003
How helpful was the post-call handover in ensuring that you were able to carry out the tasks expected of you? (Junior staff only)	31	3.93 (0.96)	63	4.19 (0.86)	.22
How helpful has the handover process been in ensuring that you were able to coordinate and manage your team and the service? (Senior staff only)	9	4.22 (0.83)	19	3.84 (0.76)	.26
**During the time period, when you received the post-call morning handover from a colleague, how often did you find that:**					
Information was missing or incorrect.	40	2.22 (0.77)	81	1.76 (0.79)	.003
Patients turned out to be sicker than expected.	40	2.10 (0.63)	81	1.60 (0.68)	<.001
Throughout the course of the day, something happened relating to patient care which you were unprepared for.	40	2.00 (0.72)	81	1.76 (0.73)	.10
Patient care was negatively impacted due to the quality of the handover received.	40	1.65 (0.73)	81	1.29 (0.58)	.01
**How would you rate the following**					
The efficiency of the handover process. An efficient handover process = maximum productivity with minimum wasted effort.	40	3.65 (1.00)	79	4.09 (0.85)	.02
The quality of information received during handover. (With *adequate* meaning of sufficient quality to allow you to effectively care for the patients being handed over.)	40	3.80 (0.75)	79	4.30 (0.57)	<.001
The safety of the handover process This refers to the level of risk the process poses to patients.	40	4.02 (0.66)	79	4.52 (0.62)	<.001
The overall handover process.	40	3.62 (1.05)	79	4.34 (0.79)	<.001

^a^ Questions are verbatim from the survey.

### Implementation Outcomes

**Figure 2.**
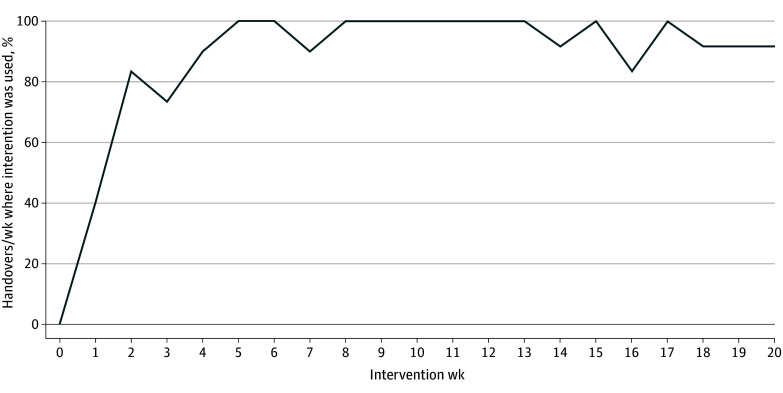
Weekly Use of the SIPS Intervention Use measured through daily recording of staff-reported use of the intervention between November 20, 2023, and April 6, 2024.

Implementation was successful and demonstrated by high rates of adoption, fidelity, and sustainability. The intervention was used during 92.9% of 211 eligible handover meetings in both sites. Weekly use reached 100% by week 5 and remained at 90% or greater for 14 of the final 15 study weeks (Figure 2). Fidelity assessment scores improved between the early (19 observations; median [IQR] score, 8 [6-9]) and full (68 observations; median [IQR] score, 9 [7-10]) implementation stages (*P* = .04) (eTable 5 in Supplement 1). Staff who used the intervention found it to be acceptable (mean [SD] score, 16.5 [3.1]), appropriate (mean [SD] score, 16.7 [2.6]), and feasible (mean [SD] score, 17.1 [2.1]). A total of 11 sustainability observations were conducted between April and June 2024. There was no significant difference in fidelity assessment scores before vs after sustainability testing (median [IQR] score, 9 [6-10] vs 7 [6-9]; *P* = .16).

## Discussion

In this cohort study, the SIPS surgical handover system, which outlines the minimum steps required for safe surgical handover, was associated with significant improvements in handover quality, patient physiology, staff-reported patient safety events, and staff-reported patient safety in 2 tertiary academic general surgical departments. Rates of adoption, fidelity, and sustainability were high and staff provided positive feedback on the acceptability, appropriateness, and feasibility of the intervention. These improvements were achieved without increasing the duration of handover meetings, suggesting minimal disruption to staff workflow.

SIPS integrates all existing evidence-based strategies and recommendations from international bodies for improving surgical handover.^8,52^ These findings align with the broader literature, which emphasizes the benefits of standardizing the handover process, highlighting critical patients,^8,11^ using a structured handover method,^28,29^ and closing the communication loop.^11^ However, the SIPS process uniquely targets the whole handover meeting,^8^ while still addressing the challenge of time pressures faced by surgical teams during morning handover.^12^

This study is one of a limited number to have reported improved patient outcomes following an intervention to improve surgical handover. Limited funding may contribute to this gap in the literature, and this study is only the second in this area to receive dedicated research funding, to our knowledge.^8^ Although several studies have reported reductions in length of stay and increased posthandover patient review, this is the first study to our knowledge to examine changes in vital signs.^8^ This is an important clinical outcome in surgical patients, given the established association between elevated Early Warning Scores and postoperative complications.^51^ In this study, patients in the intervention group demonstrated improved physiology, reflected by lower scores within 24 hours of handover, with more marked improvements over time.

To our knowledge, no previous studies of surgical handover have rigorously applied an implementation strategy to support changes,^8^ leading to uncertainty about the fidelity with which the components of a complex patient safety intervention are used and the sustainability of process changes once research team oversight ends. As a result, improvements are likely to be lost and efforts wasted.^24,53^ This study is the first to systematically apply an implementation framework to guide and support sustainable changes in this area, to our knowledge. A key success factor was early engagement with local leadership to discuss baseline findings^25^ and standardize site-specific operational aspects of the handover before introducing the intervention. This increased predictability of the handover process, which is valued by residents.^12^ Other enablers included the recruitment of key on-site support personnel, high rates of training completion, daily contact with staff, and regular performance feedback.

### Limitations

This study had several limitations, including its quasiexperimental design and retrospective manual data collection, which introduced the potential for bias. While a randomized design was considered, it was deemed inappropriate, as crossover of staff between handover periods, surgical teams, and study sites did not allow for a distinct unit of randomization (eg, team, ward, or handover meeting) without contamination between groups. This limitation is reflected in the broader literature, where few handover studies use randomization,^7,8^ and in the evaluation of the WHO Surgical Safety Checklist, the effectiveness of which has largely been demonstrated through interrupted time-series analysis.^54^ Indeed, some have argued that randomized designs may be inherently unsuitable for studying handover interventions.^55^

Prospective collection of patient outcome data may have reduced missing data, but was not feasible due to staffing constraints. Likewise, a complete electronic patient record would have improved data completeness; however, this was unavailable. The study was also conducted in 2 teaching hospitals within a single geographic region. Future research should explore the generalizability of the intervention across clinical settings and longer-term sustainability. Blinding was not possible due to the overt nature of observations, which introduced the potential for Hawthorne bias; however, the strong implementation outcomes demonstrated in this study may suggest a beneficial role for overt observation as part of an implementation strategy.

## Conclusions

In this cohort study, implementation of the SIPS surgical handover system was associated with improvements in handover quality, patient physiology, and staff-reported patient safety without prolonging handover meeting duration in 2 tertiary academic hospitals. These findings provide support for the effectiveness of the SIPS surgical handover system in reducing risk to patients during handover.

## Notes

Supplement 1. eTable 1. Strategy for implementation of the SIPS surgical handover system eTable 2. Details of outcome assessment, data sources, and time periods eTable 3. Composite fidelity score for assessing use of the SIPS intervention eTable 4. Staff experience survey demographics eTable 5. Fidelity assessment during early versus full implementation periods eAppendix 1. Research nurse observation proforma eAppendix 2. Daily staff survey eAppendix 3. Staff experience survey

Supplement 2. Members of the SURGical Improvement Network (SURGIN)

Supplement 3. Data Sharing Statement
