# Supplementary material for: A Surgical Handover System for Patient Physiology and Safety
Source: JAMA Netw Open. 2025 Oct 6;8(10):e2538896. doi: 10.1001/jamanetworkopen.2025.38896 (PMC12501811; doi:10.1001/jamanetworkopen.2025.38896)
Supplement: Supplement 1. — eTable 1. Strategy for implementation of the SIPS surgical handover system eTable 2. Details of outcome assessment, data sources, and time periods eTable 3. Composite fidelity score for assessing use of the SIPS intervention eTable 4. Staff experience survey demographics eTable 5. Fidelity assessment during early versus full implementation periods eAppendix 1. Research nurse observation proforma eAppendix 2. Daily staff survey eAppendix 3. Staff experience survey [file jamanetwopen-e2538896-s001.pdf]

Supplemental Online Content

Ryan JM, Lynn TM, Kavanagh DO, et al; SURGical Improvement Network (SURGIN). A surgical handover system for patient physiology and safety. *JAMA Netw Open*. 2025;8(10):e2538896. doi:10.1001/jamanetworkopen.2025.38896

eTable 1. Strategy for implementation of the SIPS surgical handover system
eTable 2. Details of outcome assessment, data sources, and time periods
eTable 3. Composite fidelity score for assessing use of the SIPS intervention
eTable 4. Staff experience survey demographics
eTable 5. Fidelity assessment during early versus full implementation periods
eAppendix 1. Research nurse observation proforma
eAppendix 2. Daily staff survey
eAppendix 3. Staff experience survey

This supplemental material has been provided by the authors to give readers additional information about their work.

© 2025 Ryan JM et al. *JAMA Network Open*.

eTable 1. Strategy for implementation of the SIPS surgical handover system

Stage	Exploration	Installation	Early implementation	Full implementation<br>***(Definition: All training complete and adoption rate at 100%)***
**Time period**	Pre-intervention<br>Jan – Sept 2023	Months 0–1<br>Oct – Nov 2023	Months 1–2<br>Nov – Dec 2023<br><br>Site A: 20/11/2023<br>Site B: 27/11/2023	Months 2–7<br>Dec 2023 – May 2024<br><br>Site A: 20/12/2023<br>Site B: 11/12/2023
**Activities**	National survey of practice[12]	Formation of implementation team	Staff training (didactic)	Additional training after staff rotations
	Handover ethnography[25] at both sites	Establishment of site leadership	Cognitive aids in all handover areas	Twice-monthly performance reports
		Standardization of handover process (mandatory location and time for handover, required attendees and role expectations)	In-person Just-in-Time Training and coaching for handover leaders and junior staff<br><br>Daily contact with staff	Proof of audit, training, and participation for Continuing Professional Development purposes
		Communication strategy for notification of staff	Anonymous staff channel for reporting issues	Comprehensive performance report in Feb 2024
		Site WhatsApp groups for study information and updates	Plan–Do–Study–Act cycles to manage issues	Daily contact with staff until April 2024

© 2025 Ryan JM et al. *JAMA Network Open*.

eTable 2. Details of outcome assessment, data sources, and time periods

Outcomes	Outcome measure/definition	Data source	Time period[a]
**Clinical effectiveness**[8]			
**Patient outcomes**	Change in vital signs (reflected by the EWS[53,b]) at 6, 12, and 24 hours post-handover;<br>Total hospital length of stay;<br>Transfers to higher-level care;<br>In-hospital mortality	Retrospective patient chart review, electronic patient records, the National Office of Clinical Audit – Intensive Care Unit database	Pre: 3rd May–26th Nov '23<br>Post: 11th Dec '23–5th April '24
	Handover-related patient safety events	Daily surveys of junior staff	Pre: 15th May–5th July '23<br>Post: 22nd Jan–22nd Mar '24
**Handover outcomes**	Quality of the handover process	Direct observation of handover	Pre: 9th Jan–6th April '23<br>Post: 24th Nov '23–19th June '24
**Staff outcomes**	Staff perceptions of handover safety, efficiency, and quality, measured before and after intervention	Cross-sectional staff experience survey	Pre: July '23<br>Post: Feb & Mar '24
**Implementation** [31]			
**Adoption**	Uptake of the intervention by staff	Daily staff-reported use of the intervention	20th Nov '23–6th April '24
**Fidelity**	Whether the intervention is used as directed	Fidelity score calculated from handover observations	24th Nov '23–19th June '24
**Sustainability**	Whether an intervention is used by the service after the study period	Random handover observations after the research team ceased communication with staff	8th April–19th June '24
**Acceptability, appropriateness and feasibility**	Whether staff feel the intervention is acceptable, feasible, and appropriate	Cross-sectional, validated staff survey[32] during the full implementation period. Each measure had a max total score of 20 (5 points per question) per person	Feb–Mar '24

[a] The overall pre-intervention phase ran from 9th January – 19th November 2023; the overall post-intervention phase ran from 20th November 2023 – 19th June 2024. Outcome measurement time periods varied according to patient, handover, and staff outcome categories. Implementation outcomes were not measured pre-intervention (not applicable).

[b] The Early Warning Score (EWS) is a clinical tool used to measure physiological status, calculated using patient vital sign values.[53]

© 2025 Ryan JM et al. *JAMA Network Open*.

eTable 3. Composite fidelity score for assessing use of the SIPS intervention

	Fidelity assessment item (1 point available for each item, maximum score = 10)
**1**	Sick patients discussed first (or not applicable; i.e., no sick patients for discussion)
**2**	Sick patients presented by the handover leader (or not applicable)
**3**	ISBAR used to present >/=90% of patient
**4**	Verbal priority list stated
**5**	Priority list stated after patient presentations were completed
**6**	Priority list stated by handover leader
**7**	Priority list contained all relevant information (i.e., tasks for completion, sick patients, and patients for theatre, where applicable)
**8**	Summary provided
**9**	Summary provided at end of handover meeting
**10**	Summary provided by member of receiving team

© 2025 Ryan JM et al. *JAMA Network Open*.

eTable 4. Staff experience survey demographics

	Pre-intervention	Post-intervention	Total	*p*
Total responses	40	82	122	
Hospital				0.072
Site A	18	51	69	
Site B	22	31	53	
Grade of staff				0.291
Intern	10	34	44	
Resident grades				
Senior house officer	17	20	37	
Junior registrar	4	9	13	
Senior registrar	3	6	9	
Specialist registrar	6	13	19	

eTable 5. Fidelity assessment during early versus full implementation periods

	Fidelity assessment item	Early implementation (n=19), n (%)	Full implementation (n=68), n (%)	*p*
1	Sick patients discussed first where applicable	14 (93)	53 (100)	0.058
2	Sick patients presented by the handover leader	12 (86)	51 (96)	0.14
3	ISBAR used to present >/=90% of patients	11 (58)	51 (75)	0.145
4	Verbal priority list stated	14 (74)	55 (81)	0.493
5	Priority list stated after patient presentations were completed	13 (93)	51 (93)	0.987
6	Priority list stated by handover leader	12 (86)	49 (89)	0.725
7	Priority list contained all relevant information (i.e., tasks for completion, sick patients, and patients for theatre, where applicable)	5 (36)	44 (80)	0.001*
8	Summary provided	16 (84)	62 (91)	0.378
9	Summary provided at end of handover meeting	14 (87)	53 (85)	0.836
10	Summary provided by member of receiving team	16 (100)	60 (97)	0.467

© 2025 Ryan JM et al. *JAMA Network Open*.

eAppendix 1. Research nurse observation proforma

Surgical Handover - Research nurse observational data upload

Handover logistics

Basic handover information

* 1. Name of observer:

* 2. Hospital:

- [redacted] Hospital
- [redacted] Hospital

* 3. Date of observational data:

Date

Date

DD/MM/YYYY

* 4. Did you observe a handover meeting?

Answering no will end this survey.

- Yes
- No

If no, please provide an explanation for why no handover was observed:

* 5. Handover **start** time:

Time

Time AM/PM

hh mm -

* 6. Handover **finish** time:

Time

Time AM/PM

hh mm -

© 2025 Ryan JM et al. *JAMA Network Open*.

* 7. Handover duration (minutes):
Please use whole numbers only and no words

* 8. Handover location:

* 9. Number of attendees:

* 10. Attendees present (select all that apply):

- ☐ Consultant
- ☐ Post-call senior registrar
- ☐ Post-call junior registrar (not applicable to Tallaght Hospital)
- ☐ Post-call senior house officer
- ☐ Post-call intern
- ☐ Day-shift senior registrar
- ☐ Day-shift junior registrar (not applicable to Tallaght Hospital)
- ☐ Day-shift senior house officer
- ☐ Day-shift intern
- ☐ Other (please specify if additional attendees, or multiples of any of the above were present)

11. Any important comments on the above questions can be included here:

© 2025 Ryan JM et al. *JAMA Network Open*.

Surgical Handover - Research nurse observational data upload

Handover content and structure

* 12. Were sick patients presented first?
I.e., Unstable patients, watchers, NEWS >/= 4

- ◯ Yes
- ◯ No
- ◯ Not applicable - No sick patients were mentioned during handover meeting

13. Who presented the sick patients?

* 14. Number of patient presentations given during handover meeting:

* 15. Number of patient presentations where ISBAR was used correctly:

Identity: *Patient name*
Situation: *Why are they in hospital? (e.g., presenting complaint/likely diagnosis/reason for surgical review, etc)*
Background: *History, exam, investigations, etc*
Assessment: *Clinical impression (e.g., Working diagnosis/illness severity)*
Recommendation: *Plan or actions*

* 16. Was a verbal priority list given?

- ◯ Yes
- ◯ No
- ◯ Not applicable - No patients were discussed during the handover meeting

17. Who gave the priority list?

18. When was the priority list given?

© 2025 Ryan JM et al. *JAMA Network Open*.

19. What did the priority list include?

	Yes	No	Not applicable
Sick patients	○	○	○
Patients for theatre	○	○	○
Tasks for the day	○	○	○

Other (please specify)

* 20. Was a summary given?

- ○ Yes
- ○ No
- ○ Not applicable - No patients were discussed during the handover meeting

21. Who gave the summary?

22. When was the summary given?

© 2025 Ryan JM et al. *JAMA Network Open*.

* 24. Did the interns appear to be actively listening during the meeting? *(E.g., watching the presenter, taking notes, etc.)*

- Yes
- No
- No intern was present
- Other (please specify)

* 25. Did the post-call team face the interns during the meeting? *(I.e., were the interns included in the physical space of the handover?)*

- Yes
- No
- No intern was present
- Other (please specify)

* 26. Did the interns ask any questions during the handover meeting?

- Yes
- No
- No intern was present
- Other (please specify)

27. Any important comments about the above questions can be included here:

© 2025 Ryan JM et al. *JAMA Network Open*.

eAppendix 2. Daily staff survey

© 2025 Ryan JM et al. *JAMA Network Open*.

Post-take intern survey

Information Leaflet

This survey takes 1-2 minutes to complete and relates to the day shift immediately after the on-call period. You have been sent this survey because you were involved in the care of newly admitted general surgical patients during a post-take period within the last 1-2 days.

The best time to complete this survey is after the evening ward round on the post-take day. Your answers are anonymous and will be pooled with results from another hospital.

Risk management pathways are in place in your hospital for reporting of patient safety issues.

[redacted]

[redacted]

* 1. Which post-take day does this survey relate to?

Date

Date

DD/MM/YYYY

* 2. Which hospital do you work in?

- ◯ [redacted] Hospital
- ◯ [redacted] Hospital

* 3. Are you an...

- ◯ Intern
- ◯ Senior House Officer
- ◯ Registrar
- ◯ Senior Registrar
- ◯ Specialist Registrar

Other (please specify)

© 2025 Ryan JM et al. *JAMA Network Open*.

* 4. How did you receive information about the newly admitted patients this morning?

e.g., did your Registrar sit down for a formal handover meeting with you? Phone/text you? Or were you just given the information during the morning ward round?

◯ A handover meeting before the ward round

◯ I was told about the patients on the ward round

Other (please specify)

* 5. Were any patients unintentionally missed on the morning ward round (including consults)?

i.e., At any point during the day, after the ward round was finished, the team realised that there was a patient requiring review who had not yet been seen

◯ Yes

◯ No

6. If any patients were missed on the ward round, please specify how many. You may give further detail if you wish

* 7. Did anything happen today, relating to patient care, which you were unprepared for?

◯ Yes

◯ No

* 8. During the day, did any of the newly admitted patients turn out to be sicker than expected based on the information given to you in the morning (as described above)?

◯ Yes

◯ No

* 9. Did any patient safety issues occur during the post-take day which could have been prevented or improved by the information given at the morning handover?

e.g., information given at handover was missing or incorrect, or a patient was not handed over, which led to a near miss or a patient safety event

◯ Yes

◯ No

© 2025 Ryan JM et al. *JAMA Network Open*.

Post-take intern survey

Patient safety issues

Please answer the questions below for each patient safety issue which occurred.

Remember, these issues should be related to handover in some way - i.e., the issue occurred, or was made worse, due to the information given at handover.

For example, important handover information was missing or incorrect, or a patient was not handed over.

Terms used:

1. **Near miss: An incident that was prevented from occurring due to timely intervention or chance, and which could have resulted, if it had not been prevented, in injury or harm**
2. **Negligible harm: Adverse event leading to minor injury not requiring first aid, or reduced quality of patient experience due to inadequate provision of information**
3. **Minor harm: Minor injury or illness, first aid treatment required**
4. **Moderate harm: Significant injury requiring medical treatment, no long-term or permanent effects**
5. **Major harm: Major injuries/long term incapacity or disability requiring medical treatment**
6. **Extreme harm: Incident leading to death or major permanent incapacity**

If needed, please see the following risk assessment tool for the full definitions of the different types of harm: Risk assessment tool

* 10. Issue 1

- ◯ Near miss
- ◯ Negligible or minor harm
- ◯ Moderate harm
- ◯ Major harm
- ◯ Extreme harm

11. Issue 2

- ◯ Near miss
- ◯ Negligible or minor harm
- ◯ Moderate harm
- ◯ Major harm
- ◯ Extreme harm

© 2025 Ryan JM et al. *JAMA Network Open*.

12. Issue 3

- ◯ Near miss
- ◯ Negligible or minor harm
- ◯ Moderate harm
- ◯ Major harm
- ◯ Extreme harm

13. Issue 4

- ◯ Near miss
- ◯ Negligible or minor harm
- ◯ Moderate harm
- ◯ Major harm
- ◯ Extreme harm

14. Issue 5

- ◯ Near miss
- ◯ Negligible or minor harm
- ◯ Moderate harm
- ◯ Major harm
- ◯ Extreme harm

15. You can place any comments you may have below (or additional safety issues if more than 5), please ensure not to include any identifying patient or staff details.

© 2025 Ryan JM et al. *JAMA Network Open*.

eAppendix 3. Staff experience survey

© 2025 Ryan JM et al. *JAMA Network Open*.

Staff perceptions of the surgical handover process

Section 1

* 1. Which hospital are you currently working in?
(If you changed hospitals in January, enter the hospital you worked in before January)

- ◯ [redacted] Hospital
- ◯ [redacted] Hospital

* 2. Which staff grade are you?

- ◯ Intern
- ◯ Senior House Officer
- ◯ Junior Registrar
- ◯ Senior Registrar
- ◯ Specialist Registrar

© 2025 Ryan JM et al. *JAMA Network Open*.

Staff perceptions of the surgical handover process

Section 2

* 3. How often did you attend the post-call morning handover on days that your team were post-take?

- Never (almost no handovers attended)
- Occasionally (around 25% attended)
- Sometimes (around 50%)
- Often (around 75%)
- Almost always (around 90%)

* 4. What was the **most common** reason for you **not to attend** the handover? Select one

- I was not invited, or I was told that I did not need to attend
- I was expected to attend but I was not given the time or location of the handover
- I did not want to attend
- I was busy carrying out other patient-care activities
- Other (please specify)

5. If you reported not being invited to the handover, or being told that you did not have to attend, how often did this occur? You can skip this question if not applicable

- Almost always (around 90% of your team's handovers)
- Often (around 75%)
- Sometimes (around 50%)
- Occasionally (around 25%)
- Never (almost 0% of the time)
- Not applicable

* 6. How often was the post-call handover used to **prioritise** jobs to be completed that day?

Examples of prioritisation: "These are the scans we need today in order of importance", "These are our patients for theatre today in order of urgency", "these are our sickest patients", etc.

- Almost always (around 90% of the time)
- Often (around 75%)
- Sometimes (around 50%)
- Occasionally (around 25%)
- Never (almost 0% of the time)

© 2025 Ryan JM et al. *JAMA Network Open*.

* 7. How helpful was the post-call handover in ensuring that you were able to carry out the tasks expected of you?

- ◯ Extremely helpful
- ◯ Very helpful
- ◯ Somewhat helpful
- ◯ Unhelpful
- ◯ Extremely Unhelpful

© 2025 Ryan JM et al. *JAMA Network Open*.

Staff perceptions of the surgical handover process

Section 2

* 8. Since this study began in November 2023, how helpful has the handover process been in ensuring that you were able to coordinate and manage your team and the service?

- ◯ Extremely helpful
- ◯ Very helpful
- ◯ Somewhat helpful
- ◯ Unhelpful
- ◯ Extremely Unhelpful

© 2025 Ryan JM et al. *JAMA Network Open*.

Staff perceptions of the surgical handover process

Adequacy of information received during handover

When you received the post-call morning handover from a colleague, how often did you find that:

* 9. Information was missing or incorrect

- Never (almost 0% of the time)
- Occasionally (around 25% of the time)
- Sometimes (around 50% of the time)
- Often (around 75% of the time)
- Almost always (around 90% of the time)

* 10. Patients turned out to be sicker than expected

- Never (almost 0% of the time)
- Occasionally (around 25% of the time)
- Sometimes (around 50% of the time)
- Often (around 75% of the time)
- Almost always (around 90% of the time)

* 11. Throughout the course of the day, something happened relating to patient care which you were unprepared for

- Never (almost 0% of the time)
- Occasionally (around 25% of the time)
- Sometimes (around 50% of the time)
- Often (around 75% of the time)
- Almost always (around 90% of the time)

* 12. Patient care was negatively impacted due to the quality of the handover received

- Never (almost 0% of the time)
- Occasionally (around 25% of the time)
- Sometimes (around 50% of the time)
- Often (around 75% of the time)
- Almost always (around 90% of the time)

© 2025 Ryan JM et al. *JAMA Network Open*.

Staff perceptions of the surgical handover process

Staff satisfaction with handover

How would you rate the following:

* 13. The efficiency of the handover process

An efficient handover process = maximum productivity with minimum wasted effort

- Very good (very efficient)
- Good
- Acceptable
- Poor
- Very poor (very inefficient)

* 14. The quality of information received during handover

"Adequate" = of sufficient quality to allow you to effectively care for the patients being handed over

- Very adequate
- Adequate
- Neither adequate nor inadequate
- Inadequate
- Very inadequate

* 15. The safety of the handover process

This refers to the level of risk the process poses to patients

- Almost no risk involved
- Minor level of risk
- Moderate level of risk
- Major level of risk
- Extremely risky process

* 16. The overall handover process

- Very good
- Good
- Acceptable
- Poor
- Very poor

© 2025 Ryan JM et al. *JAMA Network Open*.

Staff perceptions of the surgical handover process

Section 3

The following questions relate to your perspectives of the SIPS handover intervention.

* 17. Please indicate your agreement with the following statements regarding the **acceptability** of the SIPS handover intervention.

	Completely disagree	Disagree	Neither agree nor disagree	Agree	Completely agree
It meets my approval.	◯	◯	◯	◯	◯
It is appealing to me.	◯	◯	◯	◯	◯
I like it.	◯	◯	◯	◯	◯
I welcome its introduction.	◯	◯	◯	◯	◯

* 18. Please indicate your level of agreement regarding the **appropriateness** of the SIPS handover intervention.

	Completely disagree	Disagree	Neither agree nor disagree	Agree	Completely agree
It seems fitting.	◯	◯	◯	◯	◯
It seems suitable.	◯	◯	◯	◯	◯
It seems applicable.	◯	◯	◯	◯	◯
It seems like a good match for the department.	◯	◯	◯	◯	◯

* 19. Please indicate your agreement regarding the **feasibility** of the SIPS handover intervention.

	Completely disagree	Disagree	Neither agree nor disagree	Agree	Completely agree
It seems implementable.	◯	◯	◯	◯	◯
It seems possible.	◯	◯	◯	◯	◯
It seems doable.	◯	◯	◯	◯	◯
It seems easy to use.	◯	◯	◯	◯	◯

© 2025 Ryan JM et al. *JAMA Network Open*.
