# Supplementary material for: A Surgical Handover System for Patient Physiology and Safety
Source: JAMA Netw Open. 2025 Oct 6;8(10):e2538896. doi: 10.1001/jamanetworkopen.2025.38896 (PMC12501811; doi:10.1001/jamanetworkopen.2025.38896)
Supplement: Supplement 2. — Members of the SURGical Improvement Network (SURGIN) [file jamanetwopen-e2538896-s002.pdf]

Supplemental Online Content: Nonauthor Collaborators
*First name, last name, and suffix (if applicable) are required and will appear in PubMed.

***Group Name(s):** SURGical Improvement Network (SURGIN)

***First Name and Middle Initial(s)**	***Last Name**	***Suffix (eg, Jr, III)**	Academic Degrees	Institution	Location (city, state/province, country)	Role or Contribution, eg, chair, principal investigator	Group (if more than 1 Group listed in the byline) and/or Subgroup (eg, Steering Committee)
Cian	Hehir		MB, BCh	Beaumont Hospital	Beaumont, Dublin, Ireland	Data collector	
Tamas	TiszaiSzucs			Tallaght University Hospital	Tallaght, Dublin, Ireland	Assisted with collection of ICU data	
Catherine	Timon		MB, BCh	Tallaght University Hospital	Tallaght, Dublin, Ireland	Intern Liaison	
Shelby	Tiller		MB, BCh	Royal College of Surgeons in Ireland	Dublin, Ireland	Data collector	
Nicola	Raftery		MRCSI	Tallaght University Hospital	Tallaght, Dublin, Ireland	Secondary site lead	
Maryam	Albreiki		MB, BCh	Beaumont Hospital	Beaumont, Dublin, Ireland	Intern Liaison	
Alyssa	Clark		MB, BCh	Royal College of Surgeons in Ireland	Dublin, Ireland	Data collector	
Caoimhe	Burke		MB, BCh	Beaumont Hospital	Beaumont, Dublin, Ireland	Data collector	
Liam	Coughlan		MB, BCh	Tallaght University Hospital	Tallaght, Dublin, Ireland	Data collector	
Roseanna	Martyn			Royal College of Surgeons in Ireland	Dublin, Ireland	Data collector	
