# Supplementary material for: A Surgical Handover System for Patient Physiology and Safety
Source: JAMA Netw Open. 2025 Oct 6;8(10):e2538896. doi: 10.1001/jamanetworkopen.2025.38896 (PMC12501811; doi:10.1001/jamanetworkopen.2025.38896)
Supplement: Supplement 3. — Data Sharing Statement [file jamanetwopen-e2538896-s003.pdf]

Data Sharing Statement

Ryan. A Surgical Handover System for Patient Physiology and Safety. *JAMA Netw Open*. Published October 06, 2025. doi:10.1001/jamanetworkopen.2025.38896

Data
Data available: No
